# *Cyperus articulatus* L. (Cyperaceae) Rhizome Essential Oil Causes Cell Cycle Arrest in the G_2_/M Phase and Cell Death in HepG2 Cells and Inhibits the Development of Tumors in a Xenograft Model

**DOI:** 10.3390/molecules25112687

**Published:** 2020-06-09

**Authors:** Mateus L. Nogueira, Emilly J. S. P. de Lima, Asenate A. X. Adrião, Sheila S. Fontes, Valdenizia R. Silva, Luciano de S. Santos, Milena B. P. Soares, Rosane B. Dias, Clarissa A. Gurgel Rocha, Emmanoel V. Costa, Felipe M. A. da Silva, Marcos A. Vannier-Santos, Nállarett M. D. Cardozo, Hector H. F. Koolen, Daniel P. Bezerra

**Affiliations:** 1 Gonçalo Moniz Institute, Oswaldo Cruz Foundation (IGM-FIOCRUZ/BA), Bahia, Salvador 40296-710, Brazil; mateus.ln92@gmail.com (M.L.N.); sheila_suarez@yahoo.com.br (S.S.F.); valdeniziar@gmail.com (V.R.S.); luciano.biomed@gmail.com (L.d.S.S.); milenabpsoares@gmail.com (M.B.P.S.); rosanebd@gmail.com (R.B.D.); gurgel.clarissa@gmail.com (C.A.G.R.); 2 Metabolomics and Mass Spectrometry Research Group, Amazonas State University (UEA), Amazonas, Manaus 690065-130, Brazil; emillyjulianasales@gmail.com (E.J.S.P.d.L.); alineadriaoam@gmail.com (A.A.X.A.); 3 Department of Clinical Propaedeutics and Integrated Clinical, Faculty of Dentistry, Federal University of Bahia, Bahia, Salvador 40301-155, Brazil; 4 Department of Chemistry, Federal University of Amazonas (UFAM), Amazonas, Manaus 690065-130, Brazil; emmanoelvc@gmail.com (E.V.C.); felipesaquarema@bol.com.br (F.M.A.d.S.); 5 Oswaldo Cruz Institute, Oswaldo Cruz Foundation, Rio de Janeiro 21040-360, Brazil; marcos.vannier@ioc.fiocruz.br; 6 Amazonia Museum (MUSA), Amazonas, Manaus 69099-415, Brazil; nallarett.davila@gmail.com

**Keywords:** *Cyperus articulates*, cell death, G_2_/M arrest, HepG2, antitumor

## Abstract

*Cyperus articulatus* L. (Cyperaceae), popularly known in Brazil as “priprioca” or “piriprioca”, is a tropical and subtropical plant used in popular medical practices to treat many diseases, including cancer. In this study, *C. articulatus* rhizome essential oil (EO), collected from the Brazilian Amazon rainforest, was addressed in relation to its chemical composition, induction of cell death in vitro and inhibition of tumor development in vivo, using human hepatocellular carcinoma HepG2 cells as a cell model. EO was obtained by hydrodistillation using a Clevenger-type apparatus and characterized qualitatively and quantitatively by gas chromatography coupled to mass spectrometry (GC-MS) and gas chromatography with flame ionization detection (GC-FID), respectively. The cytotoxic activity of EO was examined against five cancer cell lines (HepG2, HCT116, MCF-7, HL-60 and B16-F10) and one non-cancerous one (MRC-5) using the Alamar blue assay. Cell cycle distribution and cell death were investigated using flow cytometry in HepG2 cells treated with EO after 24, 48 and 72 h of incubation. The cells were also stained with May–Grunwald–Giemsa to analyze the morphological changes. The anti-liver-cancer activity of EO in vivo was evaluated in C.B-17 severe combined immunodeficient (SCID) mice with HepG2 cell xenografts. The main representative substances of this EO sample were muskatone (11.6%), cyclocolorenone (10.3%), α-pinene (8.26%), pogostol (6.36%), α-copaene (4.83%) and caryophyllene oxide (4.82%). EO showed IC_50_ values for cancer cell lines ranging from 28.5 µg/mL for HepG2 to >50 µg/mL for HCT116, and an IC_50_ value for non-cancerous of 46.0 µg/mL (MRC-5), showing selectivity indices below 2-fold for all cancer cells tested. HepG2 cells treated with EO showed cell cycle arrest at G_2_/M along with internucleosomal DNA fragmentation. The morphological alterations included cell shrinkage and chromatin condensation. Treatment with EO also increased the percentage of apoptotic-like cells. The in vivo tumor mass inhibition rates of EO were 46.5–50.0%. The results obtained indicate the anti-liver-cancer potential of *C. articulatus* rhizome EO.

## 1. Introduction

Natural products are an important source of new drugs for a wide range of diseases. In the latest review of natural medicines published by DJ Newman and GM Cragg of the National Institutes of Health (United States of America), they reported that 76.4% of all new drugs approved by the FDA from 1981 to 2019 (*n* = 1881) are natural products or natural-based components [1]. In particular, some plant-derived drugs are among the most important antineoplastic agents, including the family of vinca alkaloids isolated from *Catharanthus roseus* G. Don [2], etoposide obtained by the semi-synthesis from podophyllotoxin isolated from rhizome of *Podophyllum peltatum* L. [3], and paclitaxel isolated from the bark of *Taxus brevifolia* Nutt [4].

*Cyperus articulatus* L. (Cyperaceae), popularly known in Brazil as “priprioca” or “piriprioca”, is a circa 2-meter-tall medicinal plant that grows in swampy areas and/or near riverbanks in tropical and subtropical regions [5,6]. In African and American countries, *C. articulatus* rhizomes are used in popular medical practices to treat many disorders, including infections, fevers, pain, seizures, gastrointestinal and urinary disorders, bleeding, irregular menstruation, cancer, and as an abortion agent/contraceptive [5,6,7,8,9,10,11,12]. People in the Amazon grind or suck the rhizome with water to drink. It is also sold in herbal medicine stores in the USA and South America as a fluid extract or in capsules [6].

Previous pharmacological studies with crude extracts of *C. articulatus* and its components have reported this plant as a source of anticonvulsant [13], sedative [14], antifungal [15], anti-plasmodial [16], anti-*Onchocerca* [17], antibacterial [18], antioxidant [19] and cytotoxic [19] agents. Regarding its cytotoxic properties, Kavaz et al. [19] published a preliminary study showing that *C. articulatus* rhizome essential oil (EO), collected in northern Nigeria, exhibited cytotoxicity against human breast adenocarcinoma MDA-MB-231 cells, and its chemical composition included sesquiterpenes, monoterpenes, nootkatone, 6-methyl-3,5-heptadien-2-one, retinene, nopinone, cycloeucalenol, anozol, toosendanin, furanone, ethanone and vitamin A [19]. Here, the *C. articulatus* rhizome EO, collected in the Brazilian Amazon rainforest, was studied for its chemical composition, induction of cell death in vitro and the inhibition of tumor development in vivo using human hepatocellular carcinoma HepG2 cells as a cell model.

## 2. Results

### 2.1. Chemical Analysis of Cyperus articulatus Rhizome Essential Oil

The EO recovery from rhizome of *C. articulatus* was 0.58 ± 0.04% (*w*/*w*), in which a composition dominated by terpenoids was observed (Table 1). Among the different types, monoterpenes (hydrocarbons 14.59%; oxygenated 8.29%) and sesquiterpenes (hydrocarbons 8.98%; oxygenated 47.49%) and trace of diterpenes were identified. The main representative substances of this EO sample were muskatone (11.60 ± 1.19%), cyclocolorenone (10.30 ± 1.02%), α-pinene (8.26 ± 0.74), pogostol (6.36 ± 0.88%), *α*-copaene (4.83 ± 0.45%) and caryophyllene oxide (4.82 ± 0.44%).

### 2.2. Cyperus articulatus Rhizome Essential Oil Induces Cytotoxicity in a Panel of Cancer Cell Lines

The cytotoxic activity of EO was examined against five cancer cell lines (HepG2, HCT116, MCF-7, HL-60 and B16-F10) and one non-cancerous one (MRC-5) using the Alamar blue assay after 72 h of incubation (Table 2). EO showed IC_50_ values for cancer cell lines ranging from 28.5 µg/mL for HepG2 to >50 µg/mL for HCT116, and IC_50_ value for non-cancerous MRC-5 cells of 46.0 µg/mL. Doxorubicin was used as a positive control and exhibited IC_50_ values for cancer cell lines ranging from 0.03 µg/mL for HepG2 to 0.3 µg/mL for MCF-7 and an IC_50_ value for non-cancerous MRC-5 cells of 0.2 µg/mL. 5-Fluorouracil was also used as a positive control and showed IC_50_ values for cancer cell lines ranging from 0.2 µg/mL for HepG2 to 1.8 µg/mL for MCF-7 and IC_50_ value for non-cancerous MRC-5 cells of 7.5 µg/mL. The selectivity indexes (SIs) were calculated and are shown in Table 3. The EO showed low selectivity with an SI below 2-fold for all cancer cells tested.

**Figure 1:**
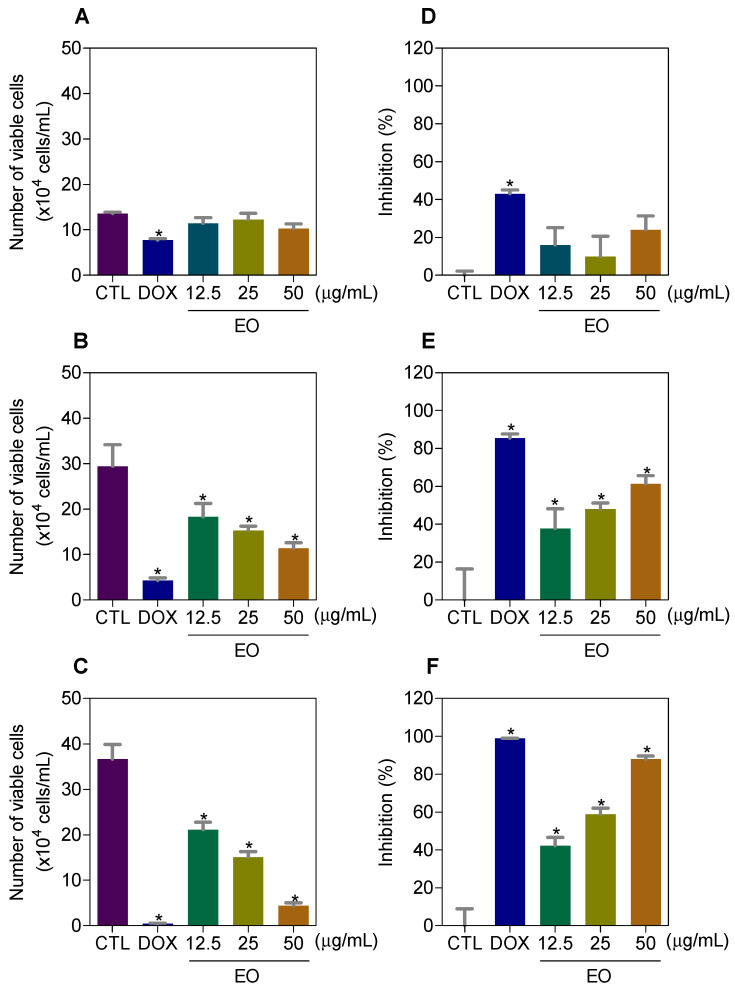
Effect of *Cyperus articulatus* rhizome essential oil (EO) on the viability of HepG2 cells, as measured by the trypan blue dye exclusion assay after 24 (**A**,**D**), 48 (**B**,**E**) and 72 (**C**,**F**) h of incubation. The negative control (CTL) was treated with a vehicle (0.5% DMSO) used to dilute EO, and doxorubicin (DOX, 1 µg/mL) was used as a positive control. The data are presented as the mean ± S.E.M. of three independent experiments carried out in duplicate. * *p* < 0.05 compared with the negative control by ANOVA, followed by the Student–Newman–Keuls test.

Then, we quantified the number of viable HepG2 cells by trypan blue exclusion (TBE) assay after 24, 48 and 72 h of incubation with EO (Figure 1). At concentrations of 12.5, 25 and 50 μg/mL, EO reduced the number of viable cells by 37.9, 47.9 and 61.4% after 48 h, and by 42.2, 58.8 and 87.9% after 72 h, respectively. Doxorubicin, at 1 μg/mL, reduced the number of viable cells by 43.0% after 24 h, 85.5% after 48 h and 98.9% after 72 h.

### 2.3. Cyperus articulatus Rhizome Essential Oil Causes Cell Cycle Arrest in the G_2_/M Phase and Cell Death in HepG2 Cells

**Figure 2:**
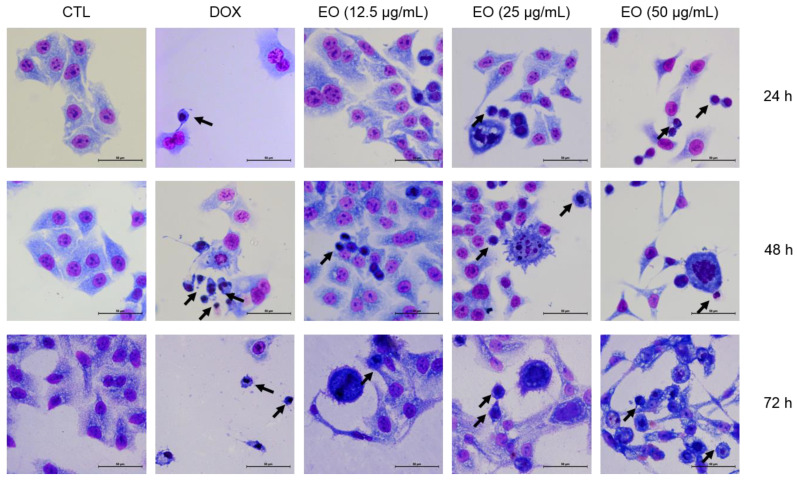
Effect of *Cyperus articulatus* rhizome essential oil (EO) on HepG2 cell morphology. The cells were stained with May-Grunwald–Giemsa and examined by optical microscopy (bar = 50 μm). The negative control (CTL) was treated with a vehicle (0.5% DMSO) used to dilute EO, and doxorubicin (DOX) was used as a positive control. The arrows indicate cell shrinkage or cells with nuclear condensation.

The morphological changes in HepG2 cells treated with EO were analyzed by optical microscopy using the May–Grunwald–Giemsa stain after 24, 48 and 72 h of incubation (Figure 2). Treatment with EO caused cell shrinkage and/or chromatin condensation, morphological changes associated to apoptotic cell death. Doxorubicin also caused morphological changes related to apoptosis.

**Figure 3:**
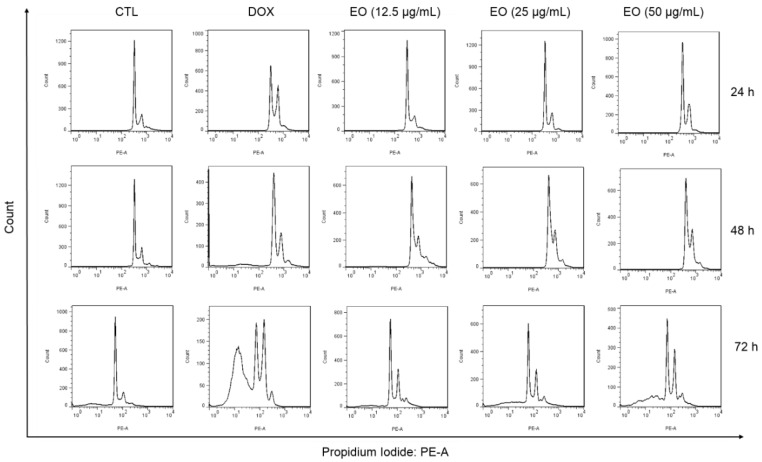
Representative flow cytometric histograms of the cell cycle distribution of HepG2 cells treated with *Cyperus articulatus* rhizome essential oil (EO). The negative control (CTL) was treated with a vehicle (0.5% DMSO) used to dilute EO, and doxorubicin (DOX, 1 µg/mL) was used as a positive control. Ten thousand events were evaluated per experiment, and cell debris was omitted from the analysis.

**Figure 4:**
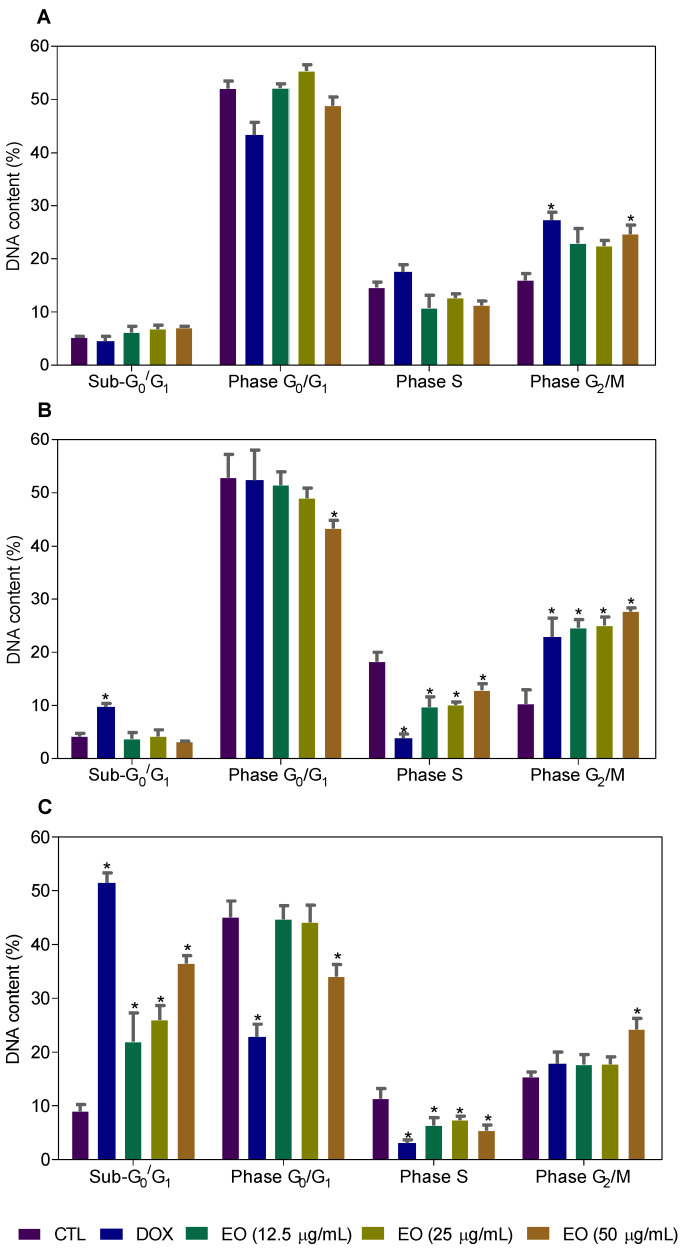
Effect of *Cyperus articulatus* rhizome essential oil (EO) on the cell cycle distribution of HepG2 cells after 24 (**A**), 48 (**B**) and 72 (**C**) h. The negative control (CTL) was treated with a vehicle (0.5% DMSO) used to dilute EO, and doxorubicin (DOX) was used as a positive control. The data are presented as the mean ± S.E.M. of three independent experiments carried out in duplicate. Ten thousand events were evaluated per experiment, and cell debris was omitted from the analysis. * *p* < 0.05 compared with the negative control by ANOVA, followed by the Newman–Keuls test.

The content of intracellular DNA in HepG2 cells treated with EO, at concentrations of 12.5, 25 and 50 μg/mL, was also quantified by flow cytometry after 24, 48 and 72 h of incubation to measure the fragmentation of internucleosomal DNA (DNA that was sub-diploid in size [sub-G_0_/G_1_]) and the distribution of cell cycle (phases G_0_/G_1_, S and G_2_/M). HepG2 cultures treated with EO showed cell cycle arrest in the G_2_/M phase, along with the fragmentation of internucleosomal DNA (Figure 3 and Figure 4). The cultures treated with EO showed 24.7, 27.7 and 24.3% of cells in the G_2_/M phase in the highest concentration after 24, 48 and 72 h of incubation, against 16.1, 10.3, 15.4 % observed for the negative control, respectively. The percentages of cells with DNA fragmentation were 22.0, 26.0 and 36.4%, at concentrations of 12.5, 25 and 50 μg/mL, respectively, against 9.0% observed for the negative control after 72 h of incubation. Doxorubicin (1 μg/mL) also caused cell cycle arrest in the G_2_/M phase, followed by the fragmentation of internucleosomal DNA.

**Figure 5:**
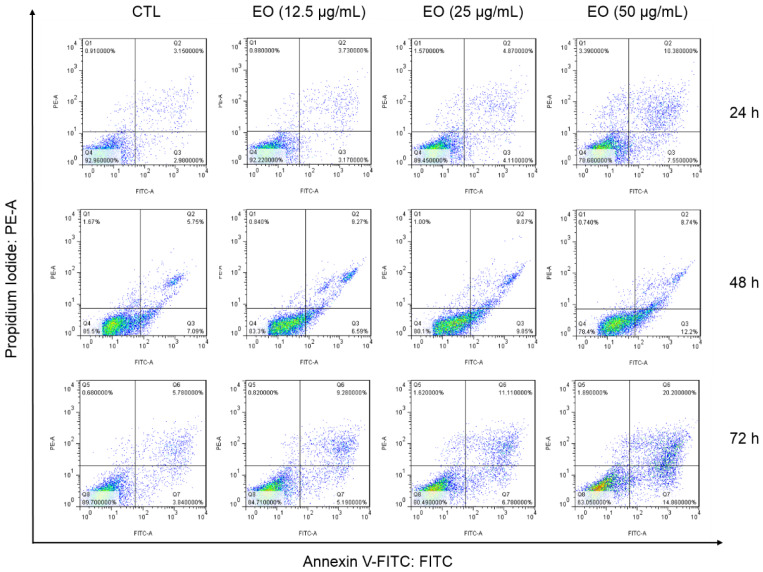
Representative flow cytometric dotplots of the cell death induction of HepG2 cells treated with *Cyperus articulatus* rhizome essential oil (EO), as measured by flow cytometry using annexin V-FITC/PI staining. The negative control (CTL) was treated with a vehicle (0.5% DMSO) used to dilute EO, and doxorubicin (DOX, 1 µg/mL) was used as a positive control. Ten thousand events were evaluated per experiment, and cell debris was omitted from the analysis.

**Figure 6:**
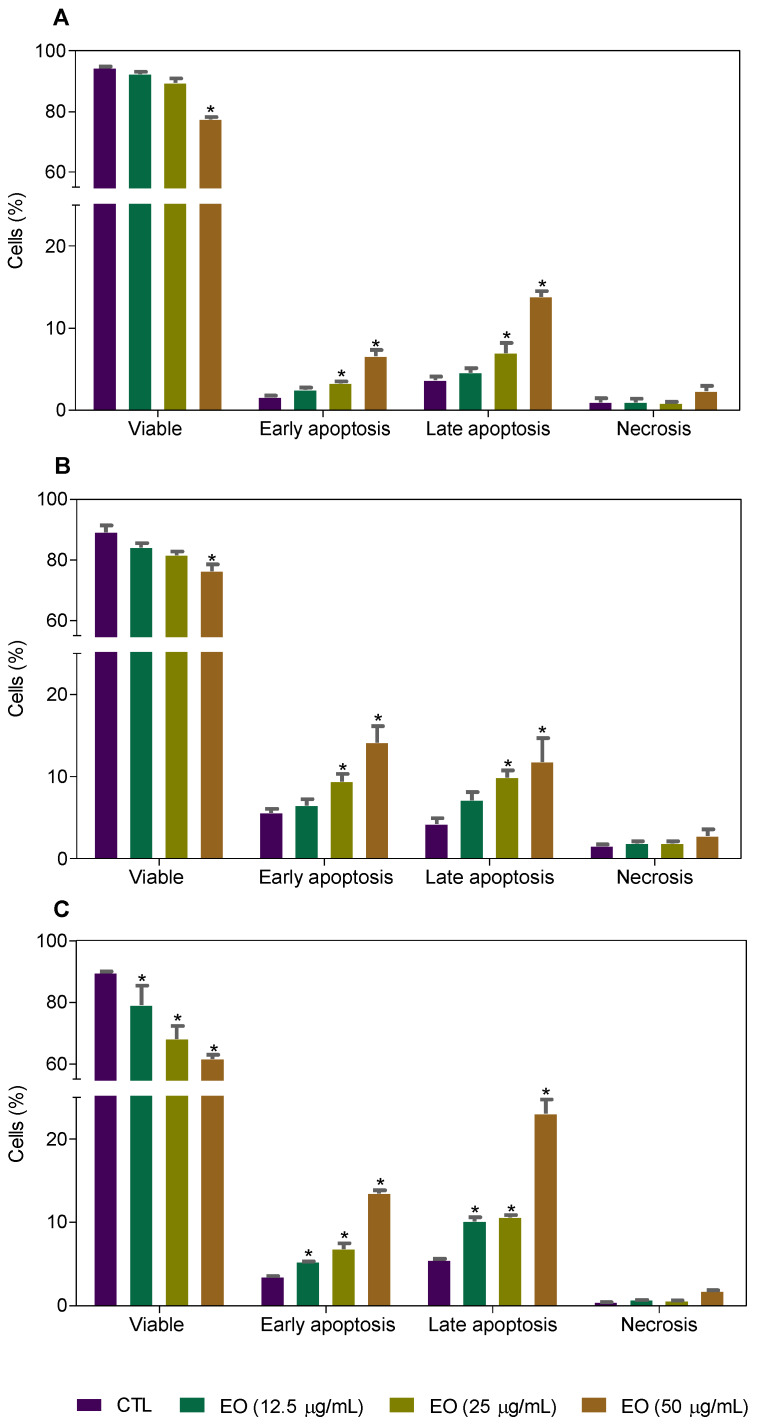
Effect of *Cyperus articulatus* rhizome essential oil (EO) on the induction of cell death in HepG2 cells, as measured by flow cytometry using annexin V-FITC/PI staining. The negative control (CTL) was treated with a vehicle (0.5% DMSO) used to dilute EO, and doxorubicin (DOX, 1 µg/mL) was used as a positive control. Ten thousand events were evaluated per experiment, and cell debris was omitted from the analysis. The data are presented as the mean ± S.E.M. of three independent experiments carried out in duplicate. * *p* < 0.05 compared with the negative control by ANOVA, followed by the Student–Newman–Keuls test.

Cell death was also evaluated by flow cytometry using annexin V-FITC/propidium iodide (PI) staining and the percentage of cells in viable (annexin V-FITC/PI double negative cells), early apoptotic (annexin V-FITC positive, but PI negative cells), late apoptotic (annexin V-FITC/PI double positive cells) and necrotic stages (PI positive, but annexin V-FITC negative cells) were quantified after 24 h, 48 h and 72 h of incubation (Figure 5 and Figure 6). Treatment with EO increased the percentage of early and late apoptotic cells.

### 2.4. Cyperus articulatus Rhizome Essential Oil Inhibits Tumor Development in a Xenograft Model

**Figure 7:**
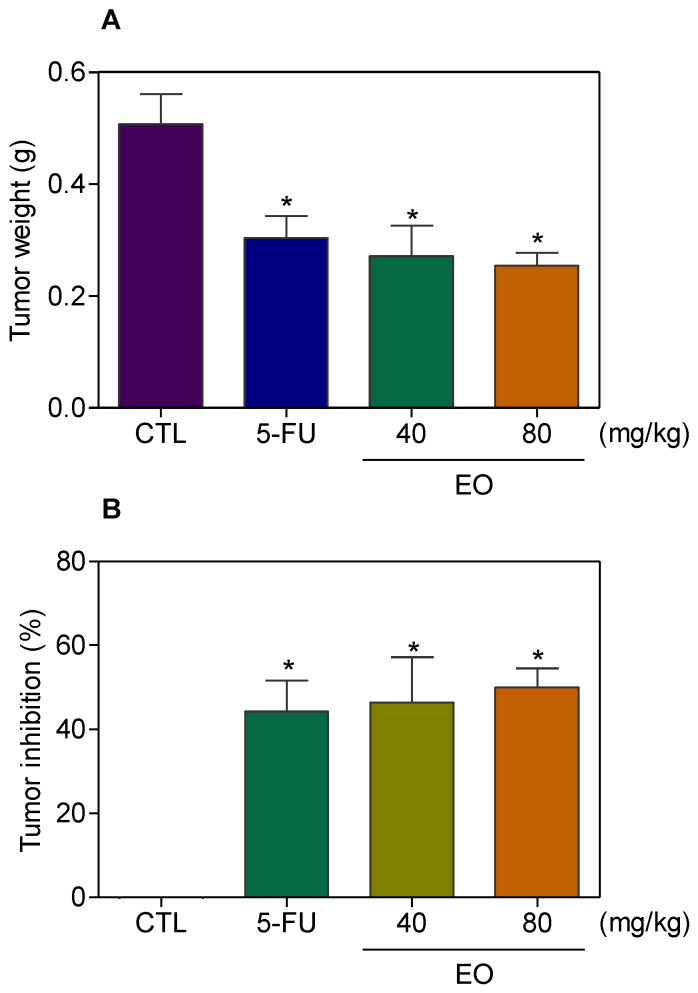
In vivo anti-liver-cancer effect of *Cyperus articulatus* rhizome essential oil (EO) in C.B-17 severe combined immunodeficient (SCID) mice with HepG2 cell xenografts. (**A**) Tumor weight (g) after treatment. (**B**) Tumor inhibition (%) after treatment. The negative control (CTL) was treated with a vehicle (5% DMSO) used to dilute EO, and 5-fluorouracil (5-FU, 10 mg/kg) was used as a positive control. Beginning 1 day after tumor implantation, the animals were treated intraperitoneally for 21 consecutive days. The data are presented as the mean ± S.E.M. of 10–20 animals. * *p* < 0.05 compared to the negative control by ANOVA, followed by the Student–Newman–Keuls test.

**Figure 8:**
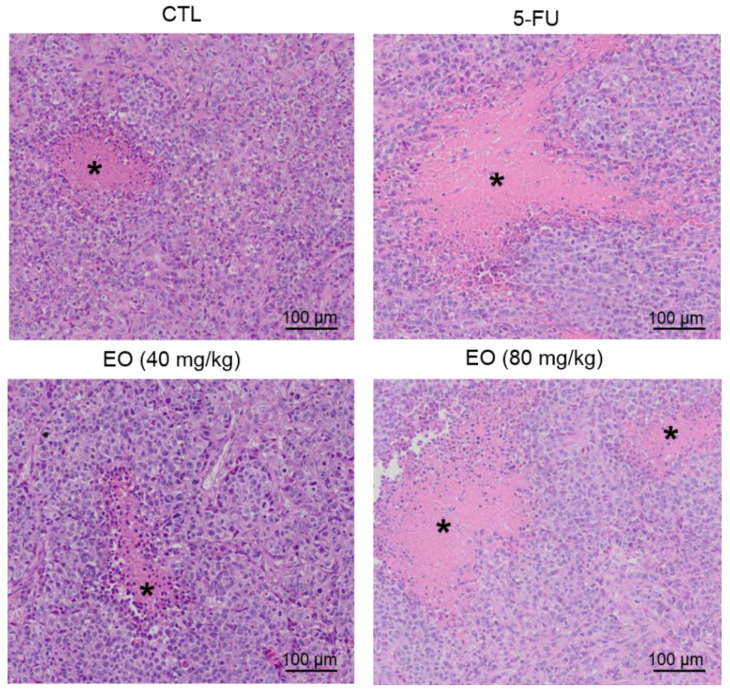
Representative histological analysis of tumors stained with hematoxylin and eosin and analyzed by optical microscopy. The asterisks indicate areas of necrosis. The negative control (CTL) was treated with a vehicle (5% DMSO) used to dilute EO, and 5-fluorouracil (5-FU, 10 mg/kg) was used as a positive control. Beginning 1 day after tumor implantation, the animals were treated intraperitoneally for 21 consecutive days.

The in vivo anti-liver-cancer activity of EO was evaluated in C.B-17 severe combined immunodeficient (SCID) mice with HepG2 cell xenografts. EO was administrated at doses of 40 and 80 mg/kg intraperitoneally, once a day, for 21 consecutive days. These doses were selected based on previous studies, using EO in tumor-bearing mice models [20,21]. The animals treated with EO showed an average tumor mass weight of 0.27 ± 0.05 g and 0.25 ± 0.02 g in the lowest and highest doses, respectively, while 0.51 ± 0.05 g was observed in the negative control group (Figure 7A). 5-Fluorouracil (10 mg/kg) was used as a positive and had an average tumor mass weight of 0.30 ± 0.04 g. The rates of tumor inhibition were 46.5–50.0 % (*p* < 0.05) for EO (Figure 7B). 5-Fluorouracil caused a tumor mass inhibition rate of 44.2 %. In the histological analysis of the tumors, we observed a carcinoma organized in multiple hypovascularized nodules delimited by a fibrous capsule, composed of hyperchromatic and highly malignant pleomorphic cells, in all groups. The tumor cells were actively dividing with the visible necrotic area observed in all groups, despite a lower frequency of mitoses in mice treated with EO at the highest dose. Degeneration and necrosis were observed in all groups, but to a lesser extent in the negative control and EO (lowest dose) groups (Figure 8).

Body and organ weight (liver, kidney, lung and heart) and hematological analyses of peripheral blood from C.B-17 SCID mice with HepG2 cell xenografts treated with EO were measured. Interestingly, no significant changes were observed in body and organ weight (*p* > 0.05) (Table 4) or hematological parameters (*p* > 0.05) (Table 5) in any group.

**Figure 9:**
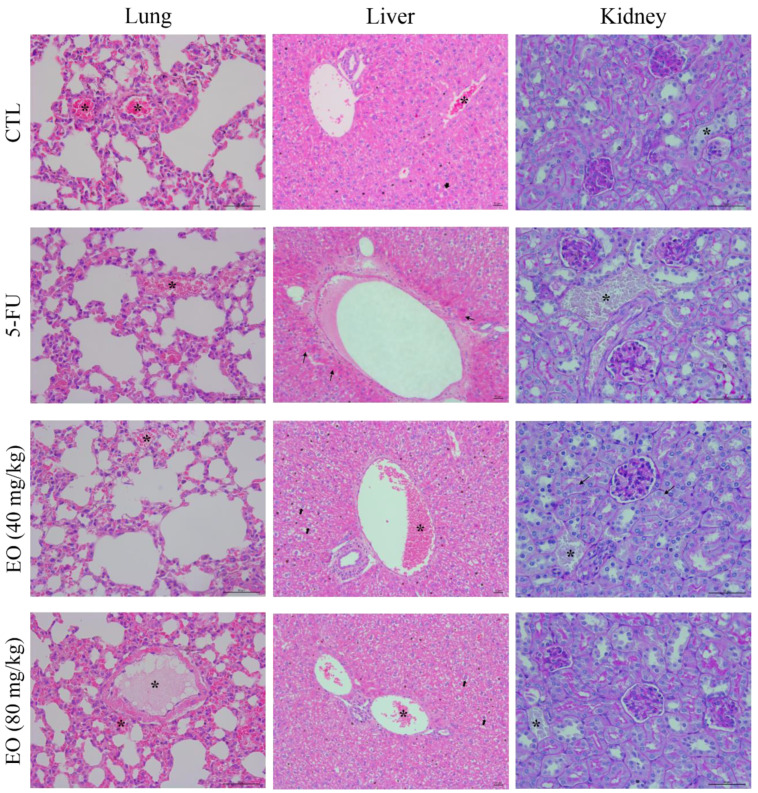
Representative histological analysis of the lungs, livers and kidneys of the C.B-17 SCID mice with HepG2 cell xenografts treated with *Cyperus articulatus* rhizome essential oil (EO). Lungs and livers were stained with hematoxylin and eosin, and kidneys were stained with Periodic acid-Schiff, and all slides were analyzed by light microscopy. The negative control (CTL) was treated with a vehicle (5% DMSO) used to dilute EO. 5-Fluorouracil (5-FU, 10 mg/kg) was used as a positive control. Beginning 1 day after tumor implantation, the animals were treated intraperitoneally for 21 consecutive days. Asterisks indicate the areas of vascular congestion, thin arrows indicate coagulation necrosis and thick arrows represent hydropic degeneration.

Histological analyses of the liver, kidney, lung and heart were also performed (Figure 9). The hepatic and portal architecture were preserved in most livers, except for two animals of the EO group (highest dose) and all animals of the 5-fluorouracil (5-FU) group, for presenting the focal areas of coagulation necrosis. The histopathological changes observed were vascular congestion, hydropic degeneration and focal inflammation, predominantly mononuclear, in the portal region, ranging from mild to moderate. In the kidneys, the tissue architecture was maintained, however, histopathological changes were observed in all experimental groups, such as moderate vascular congestion and the thickening of the basal membrane of the renal glomerulus, varying from mild to moderate, with decreased urinary space. In addition, focal areas of coagulation necrosis were observed in two animals treated with EO at the lowest dose.

The architecture of the pulmonary parenchyma was partially maintained in all groups and there was a thickening of the alveolar septum with a decrease in air space, varying from mild to moderate, in all experimental groups. Significant inflammation, predominantly of mononuclear cells, edema, congestion and hemorrhage were frequently observed, ranging from mild to severe. It is important to note that histopathological changes were more evident in the lungs of mice treated with EO at the highest dose. No histopathological changes were observed in the hearts of all animals in this study (data not shown).

## 3. Discussion

Herein, we demonstrate, for the first time, that *C. articulatus* rhizome EO causes cell cycle arrest in the G_2_/M phase and cell death in HepG2 cells and inhibits the development of tumors in a xenograft model. The main chemical constituents present in this EO sample were muskatone, cyclocolorenone, α-pinene, pogostol, α-copaene and caryophyllene oxide. The compositional results obtained are in accordance with the previously published EO of *C. articulatus* rhizome collected in the state of Pará, Brazil [5,22]. On the other hand, the same species, when collected in African countries (e.g., Egypt), presented different main compositions, including γ-patchoulene, caryophyllene oxide and α-cadinol [23], indicating a high variability in the composition of EO. Water stress, nutrition, soil and climate conditions, and other abiotic factors can be responsible for these variations.

In our cytotoxic screening program, extract/EO samples with an IC_50_ value below 30 μg/mL against cancer cell lines are selected for further studies [24,25,26,27,28]. *C. articulatus* rhizome EO showed an IC_50_ value of 28.5 μg/mL against liver cancer HepG2 cells, presenting moderate potency when compared to doxorubicin that showed an IC_50_ value of 0.03 μg/mL and 5-fluorouracil with an IC_50_ value of 0.2 μg/mL for this cell line. As mentioned above, *C. articulatus* rhizome EO, collected in northern Nigeria, showed cytotoxicity against human breast adenocarcinoma MDA-MB-231 cells [19]. Here, we demonstrate the cytotoxicity of *C. articulatus* rhizome EO, collected in the Brazilian Amazon rainforest, against five cancer cell lines (HepG2, HCT116, MCF-7, HL-60 and B16-F10) and one non-cancerous one (MRC-5). Interestingly, many of the main chemical constituents found in Nigeria’s EO sample are not found in the Brazilian EO sample.

In the *Cyperus* genus, Kilani et al. [29] reported that EO from the tuber of *C. rotundus* caused cytotoxicity in L1210 leukemia cell line with an IC_50_ value of 49 μg/mL and increased apoptotic DNA fragmentation. The main constituents identified were α-cyperone and cyperene. In another study, the EO from rhizomes of *C. rotundus* showed α-cyperone, cyperene and α-selinene as major components and was cytotoxic to human neuroblastoma SH-SY5Y cells with an IC_50_ value 1000 μg/mL [30]. The EO of the entire plant of *C. longus* induced cell death in human prostate adenocarcinoma PC3 (IC_50_ = 39.91 μg/mL) and MCF-7 (IC_50_ = 31.35 μg/mL) cell lines, and β-himachalene, α-caryophyllene oxide, irisone, β-caryophyllene oxide, humulene oxide, viridiflorol, aristolone and longiverbenone were the main chemical constituents found [31].

In relation to the cytotoxic study with the main chemical constituents of *C. articulatus* rhizome EO, the cytotoxic potential of α-pinene, α-copaene and caryophyllene oxide were previously reported [32,33,34,35,36]. α-Pinene, at 1000 μg/mL, inhibited the human carcinoma hepatocellular BEL-7402 cells proliferation, arresting cell growth in the G_2_/M phase of the cell cycle, decreasing gene and protein expressions of Cdc25C, and reducing the cycle dependence on kinase 1 (CDK1) activity [32,33]. Moreover, α-pinene (20 μg/mL) inhibited the progression of cell cycle in the G_2_/M phase and induced apoptotic cell death through the activation of caspase in human ovarian carcinoma PA-1 cells [34]. α-Copaene has been reported to suppress the proliferation of the normal neuron and N2a neuroblastoma cells of rats with an IC_50_ value of 400 μg/mL [35]. Caryophyllene oxide, at a concentration of 10 μg/mL, induced apoptosis in the PC-3 and MCF-7 cell lines by activating reactive oxygen species (ROS)-mediated mitogen-activated protein kinase (MAPK) and inhibiting the constitutive cascades of PI3K/AKT/mTOR/S6K1 signaling [36]. Cytotoxicity data for muskatone, cyclocolorenone and pogostol were not found. On the other hand, the mixture of the main and minor constituents of *C. articulatus* rhizome EO must be responsible for its cytotoxicity, since α-pinene, α-copaene and caryophyllene oxide represent less than 20% of this EO.

In addition, *C. articulatus* rhizome EO caused the cell cycle arrest in the G_2_/M phase and cell death in HepG2 cells, suggesting that this oil acts causing interruptions in the progress of the cell cycle. The EO from *Pinus koraiensis* (Pinaceae) also arrested the cell cycle in the G_2_/M phase and induced cell death in gastric adenocarcinoma MGC-803 cells. The main chemical constituents were α-pinene, limonene and β-pinene [37]. The EO from the leaves of *Melaleuca alternifolia* (Myrtaceae) arrested the cell cycle in the G_2_/M phase, causing cell death in human melanoma A-375 cells and squamous cell carcinoma HEp-2 cells [38].

Furthermore, *C. articulatus* rhizome EO inhibited the development of liver cancer HepG2 cells in a mouse xenograft model. At doses of 40 and 80 mg/kg intraperitoneally for 21 consecutive days, the inhibition rates of tumor mass were 46.5–50.0%. In addition, no significant side effects were found in this in vivo study, suggesting that, although this EO has shown low selectivity with SI below 2-fold for all cancer cells (compared to doxorubicin with an SI greater than 7-fold and 5-fluorouracil with an SI greater than 4-fold), it appears to be safe for systemic administration. The amount of EO obtained was a limitation to test it in different cell models, as well as to obtain a dose–response curve in vivo using a larger number of doses. Likewise, *Guatteria megalophylla* leaf essential also inhibited the development of HL-60 cells in a xenograft model with tumor mass inhibition rates of 16.6-48.8% at doses of 50 and 100 mg/kg, when administrated intraperitoneally once a day for nine consecutive days [28]. In addition, Chen et al. [33] demonstrated that 200 μL of α-pinene (2.67 mL/kg) injected subcutaneously for 14 ten days every two days inhibited 69.1% of tumor development in nude mice with BEL-7402 cells [33].

## 4. Material and Methods

### 4.1. Plant Material

The rhizomes of *C. articulatus* species were collected in April 2014 from the preserved green area of a private farm owned by H.H.F. Koolen located in the municipality of Rio Preto da Eva, state of Amazonas, Brazil (2° 43′ 11.2” S e 59° 31′ 08.5” W). The confirmation of the plant’s authenticity was carried out at the Herbarium of the National Institute of Research of the Amazon (INPA) in comparison to a previously deposited voucher (#24945). This work was registered (SISGEN: A50A14E) and carried out under Brazilian laws to access genetic resources.

### 4.2. Essential Oil Extraction

After collection, the fresh plant material was extracted directly by hydrodistillation with a Clevenger-type apparatus. For this, 750 g of crushed material was extracted for a period of 4 h in 3000 mL of ultrapure water (18.2 MΩ). Then, the obtained EO was extracted three times with chloroform, dried over anhydrous Na_2_SO_4_ and filtered through a nylon membrane (pore size 0.22 μm, Whatman, Maidstone, UK).

### 4.3. Chemical Analysis

The identification of chemical constituents was evaluated by gas chromatography coupled to mass spectrometry (GC-MS) with a model equipment GCMS/QP2010 Plus (Shimadzu, Kyoto, Japan) equipped with a capillary column Rtx-5 MS (30 m × 0.25 mm × 0.25 μm, Restek). Helium at a flow of 1.02 mL/min was the carrier, and injections (1 μL) were performed with 1.5 mg/mL EO stock solution in chloroform with a 1:50 partition ratio. The column temperature program was 50 to 285 °C with gradual increases of 3 °C/min. The injector and ion source temperatures were 215 and 265 °C, respectively. First, constituent identifications were based on comparisons of the spectra obtained with those stored in the Wiley 8th edition library (only similarities > 90% were considered) [39]. Confirmations were performed by calculating of the retention indices (RI) according to the equation of Van den Dool and Kratz [40] compared to a homologous series consisting by linear hydrocarbons ranging from *n*-C_7_ to *n*-C_30_. A semi-quantitative analysis was performed to obtain the relative quantity of each EO component. For that, gas chromatography with flame ionization detection (GC-FID) was applied. A system consisting of a GC2010 device (Shimadzu, Kyoto, Japan) equipped with an Rtx-5 capillary column was used under the same conditions as the GC-MS analysis. Relative quantities (%) were calculated in relation to the total chromatogram area of three independent replications.

### 4.4. In Vitro Cytotoxicity

#### 4.4.1. Cells

Cell lines HepG2 (human hepatocellular carcinoma), HCT116 (human colon carcinoma), MCF-7 (human breast adenocarcinoma), HL-60 (human promyelocytic leukemia), B16-F10 (mouse melanoma) and MRC-5 (fibroblast from human lung) were obtained from the American Type Culture Collection (ATCC, Manassas, VA, USA) and were cultured following the instructions in the ATCC animal cell culture guide. All cell lines were tested for mycoplasma using a mycoplasma stain kit (Sigma-Aldrich) to validate the use of contamination-free cells. Cell viability was examined by the trypan blue exclusion (TBE) assay in all experiments. All experiments were carried out in an exponential growth phase.

#### 4.4.2. Cytotoxicity Assay

Cell viability was quantified by the Alamar blue assay as previously described [41,42,43]. Briefly, cells were seeded in 96-well plates. EO (in eight different concentrations ranging from 0.4 to 50 μg/mL), dissolved in dimethyl sulfoxide (DMSO, Vetec Química Fina Ltd.a, Duque de Caxias, RJ, Brazil), was added to each well and incubated for 72 h. Doxorubicin (in eight different concentrations ranging from 0.04 to 5 μg/mL) (purity ≥ 95%, doxorubicin hydrochloride, Laboratory IMA S.A.I.C., Buenos Aires, Argentina) and 5-fluorouracil (in eight different concentrations ranging from 0.2 to 25 μg/mL) (5-FU, purity > 99%; Sigma Chemical Co., St Louis, MO, USA) were used as positive controls. At the end of the treatment, 20 μL of a stock solution (0.312 mg/mL) of resazurin (Sigma-Aldrich Co., Saint Louis, MO, USA) was added to each well. Absorbances at 570 and 600 nm were measured using a SpectraMax 190 Microplate Reader (Molecular Devices, Sunnyvale, CA, USA). The values of half-maximum inhibitory concentration (IC_50_) and their respective 95% confidence intervals were calculated by non-linear regression through concentration–response curves. The selectivity index (SI) was calculated using the following formula: SI = IC_50_ (non-cancerous cells)/IC_50_ (cancer cells).

#### 4.4.3. Trypan Blue Exclusion Assay

The number of viable cells and non-viable (stained with trypan blue) was determined by TBE assay. In summary, 10 μL of trypan blue (0.4%) was added to 90 μL of cell suspension and cells were counted using a Neubauer chamber.

#### 4.4.4. Internucleosomal DNA Fragmentation and Cell Cycle Distribution

The fragmentation of internucleosomal DNA and the distribution of the cell cycle were carried out as previously described [44]. Briefly, the cells were harvested in a permeabilization solution (0.1% triton X-100, 2 µg/mL PI, 0.1% sodium citrate and 100 µg/mL RNAse, all from Sigma-Aldrich Co.) and incubated in the dark for 15 min at room temperature. Then, cell fluorescence was measured by flow cytometry. At least 10^4^ events were recorded per sample using a BD LSRFortessa cytometer along with BD FACSDiva Software (BD Biosciences, San Jose, CA, EUA) and Flowjo Software 10 (Flowjo LCC, Ashland, OR, USA). Cell debris was omitted from the analyses.

#### 4.4.5. May-Grunwald-Giemsa Staining

The cells were cultured on a coverslip and stained with May–Grunwald–Giemsa. The morphological changes were examined by optical microscopy (Olympus BX41) using Image-Pro software (Media Cybernetics).

#### 4.4.6. Cell Death Quantification

The FITC Annexin V Apoptosis Detection Kit I (BD Biosciences, San Jose, CA, USA) was used to quantify cell death, and analyses were performed according to the manufacturer′s instructions. Cell fluorescence was measured by flow cytometry, as described above.

### 4.5. In Vivo Antitumor Study

#### 4.5.1. Animals

Fifty C.B-17 severe combined immunodeficient (SCID) mice (females, 25–30 g) were obtained and kept at the animal facilities of the Gonçalo Moniz Institute-FIOCRUZ (Salvador, Bahia, Brazil). The animals were housed in cages with free access to food and water. All animals were subjected to a 12:12 h light–dark cycle (lights on at 6:00 a.m.). The animal ethics committee of the Gonçalo Moniz Institute-FIOCRUZ approved the experimental protocol used (number #06/2015).

#### 4.5.2. Human Hepatocellular Carcinoma Xenograft Model

Human hepatocellular carcinoma xenograft model was performed as previously described [45,46,47]. In summary, HepG2 cells (10^7^ cells per 500 µL) were implanted subcutaneously in the left frontal axils of the mice. At the beginning of the experiment, the mice were randomly divided into four groups. Group 1: animals received injections of vehicle with 5% DMSO solution (*n* = 20). Group 2: animals received injections of 5-fluorouracil (10 mg/kg, Sigma-Aldrich, *n* = 10). Group 3: animals received injections of EO at 40 mg/kg (*n* = 10). Group 4: animals received injections of EO at 80 mg/kg (*n* = 10). Beginning one day after tumor implantation, the animals were treated intraperitoneally for 21 consecutive days. One day after the end of the treatment, the animals were anesthetized (50 mg/kg thiopental) and peripheral blood samples were collected from the brachial artery. The animals were euthanized by anesthetic overdose (100 mg/kg thiopental) and the tumors were excised and weighed.

#### 4.5.3. Toxicological Analysis

The animals were weighed at the beginning and the end of the experiment to assess the toxicological effects. All animals were observed for signs of abnormality throughout the study. Hematological analyses were performed using an Advia 60 hematology system (Bayer, Leverkusen, Germany). Livers, kidneys, lungs and hearts were collected, weighed and examined for signs of thick lesion formation, color change and/or hemorrhage. After macroscopic examination, the tumors, livers, kidneys, lungs and hearts were fixed in 4% buffered formalin and embedded in paraffin. The tissue sections were stained with hematoxylin and eosin (and Periodic acid-Schiff for kidneys slides), and a pathologist performed the analysis under an optical microscope.

### 4.6. Statistical Analysis

Data were presented as mean ± S.E.M. or as IC_50_ values with 95% confidence intervals obtained by nonlinear regressions. The differences between the experimental groups were compared through analysis of variance (ANOVA) followed by the Student–Newman–Keuls test (*p* < 0.05). All statistical analyses were performed using the GraphPad Prism (Intuitive Software for Science; San Diego, CA, USA).

## 5. Conclusions

In summary, *C. articulatus* rhizome EO presents muskatone, cyclocolorenone, *α*-pinene, pogostol, α-copaene and caryophyllene oxide as the main chemical constituents. In addition, this oil causes cell cycle arrest in the G_2_/M phase and cell death in HepG2 cells and inhibits tumor development in a xenograft model. The results obtained indicate the anti-liver-cancer potential of *C. articulatus* rhizome EO. Future studies should be directed to this EO to clarify the mechanism of action and toxicological aspects to develop *C. articulatus* as a new herbal medicine.

## Figures and Tables

**Table 1:** Chemical composition of *Cyperus articulatus* rhizome essential oil (EO).

Peak Number	Compound	Retention Time (min)	RI	ProportionalArea (%)
1	α-Pinene	5.50	931	8.26 ± 0.74
2	Verbenene	6.05	967	0.44 ± 0.06
3	β-Pinene	6.73	975	4.54 ± 0.52
4	*p*-Cymene	8.31	1025	0.42 ± 0.05
5	Limonene	8.50	1028	0.93 ± 0.11
6	Isopinocarveol	13.1	1160	2.13 ± 0.20
7	β-Phellandren-8-ol	13.5	1163	0.32 ± 0.02
8	α-Phellandren-8-ol	14.4	1168	0.81 ± 0.10
9	Terpinen-4-ol	14.9	1174	0.22 ± 0.05
10	α-Terpineol	15.5	1190	0.63 ± 0.03
11	Myrtenol	15.8	1198	3.47 ± 0.37
12	Verbenone	16.4	1205	0.71 ± 0.08
13	α-Copaene	24.6	1375	4.83 ± 0.45
14	β-Elemene	25.5	1394	0.35 ± 0.02
15	α-Gurjunene	25.8	1409	1.55 ± 0.17
16	β-Caryophyllene	26.1	1435	1.03 ± 0.11
17	β-Copaene	27.8	1440	1.22 ± 0.12
18	Unknown	28.9	-	0.84 ± 0.05
19	Unknown	29.9	-	0.15 ± 0.01
20	Caryophyllene oxide	30.7	1550	4.82 ± 0.44
21	Unknown	31.4	-	1.51 ± 0.11
22	β-Copaen-4-α-ol	32.3	1570	4.74 ± 0.40
23	Unknown	37.7	-	0.72 ± 0.04
24	Unknown	38.3	-	1.82 ± 0.15
25	Spathulenol	39.0	1588	3.68 ± 0.38
26	Unknown	43.8	-	0.43 ± 0.05
27	Globulol	44.9	1591	2.72 ± 0.29
28	Unknown	45.1	-	0.90 ± 0.08
29	Unknown	45.5	-	0.58 ± 0.06
30	Muskatone	46.0	1681	11.60 ± 1.19
31	Cyperol	46.5	1684	1.84 ± 0.15
32	Unknown	46.9	-	0.92 ± 0.09
33	Pogostol	47.2	1687	6.36 ± 0.88
34	Unknown	47.4	-	1.60 ± 0.21
35	Unknown	47.7	-	1.02 ± 0.10
36	Unknown	48.0	-	1.30 ± 0.16
37	(*E*,*E*)-Farnesol	48.1	1740	1.43 ± 0.15
38	Cyclocolorenone	48.4	1753	10.30 ± 1.02
39	Unknown	49.3	-	0.21 ± 0.02
40	Unknown	49.5	-	0.49 ± 0.05
41	Unknown	50.2	-	0.19 ± 0.01
42	(*E*)-Isogeraniol	51.6	1817	0.72 ± 0.04
	Σ_hydrocarbon monoterpenes_			14.59
	Σ_oxygenated monoterpenes_			8.29
	Σ_hydrocarbon sesquiterpenes_			8.98
	Σ_oxygenated sesquiterpenes_			47.49
	Σ_total identified_			80.07

RI: Retention Index.

**Table 2:** In vitro cytotoxicity of *Cyperus articulatus* rhizome essential oil (EO).

Cell Lines	Histological Type	IC_50_ (95% CI) in µg/mL		
		EO	DOX	5-FU
**Cancer cells**				
HepG2	Human hepatocellular carcinoma	28.5 (23.8–36.4)	0.03 (0.01–0.2)	0.2 (0.1–0.4)
HCT116	Human colon carcinoma	>50	0.1 (0.1–0.2)	0.5 (0.3–1.1)
MCF-7	Human breast adenocarcinoma	36.7 (26.7–50.5)	0.3 (0.2–0.4)	1.8 (0.7–2.3)
HL-60	Human promyelocytic leukemia	33.51 (27.3–41.2)	0.04 (0.02–0.08)	1.6 (1.2–2.2)
B16-F10	Mouse melanoma	39.7 (32.1–49.0)	0.2 (0.2–0.2)	0.5 (0.3–0.8)
**Non-cancerous cell**				
MRC-5	Human lung fibroblast	46.0 (39.6–53.6)	0.2 (0.1–0.5)	7.5 (5.2–11.0)

The data are presented as IC_50_ values, in μg/mL, with their respective 95% confidence interval (95% CI) obtained by nonlinear regression from three independent experiments carried out in duplicate, as measured by the Alamar blue assay after 72 h of incubation. Doxorubicin (DOX) and 5-fluorouracil (5-FU) were used as positive controls.

**Table 3:** Selectivity index of *Cyperus articulatus* rhizome essential oil (EO)**.**

Cancer Cells	Non-Cancerous Cells		
	MRC-5		
	EO	DOX	5-FU
HepG2	1.6	69.7	37.5
HCT116	n.d.	16.1	15
MCF-7	1.3	7.5	4.2
HL-60	1.4	52.3	4.7
B16-F10	1.2	11.0	15

The data are presented as the selectivity index (SI) calculated using the following formula: SI = IC_50_ (non-cancerous cells)/IC_50_ (cancer cells). Doxorubicin (DOX) and 5-fluorouracil (5-FU) were used as positive controls. n.d. = not determined.

**Table 4:** Effect of *Cyperus articulatus* rhizome essential oil (EO) on body and relative organ weight from C.B-17 SCID mice with HepG2 cell xenografts.

Parameters	CTL	5-FU	EO	
**Dose (mg/kg)**	-	10	40	80
Survival	20/20	10/10	10/10	10/10
Initial body weight (g)	21.4 ± 0.5	19.6 ± 0.6	21.0 ± 0.4	21.0 ± 0.5
Final body weight (g)	22.1 ± 0.5	20.5 ± 0.5	19.8 ± 0.9	21.9 ± 0.5
Liver (g/100 g body weight)	4.8 ± 0.2	4.8 ± 0.2	5.4 ± 0.3	4.9 ± 0.3
Kidney (g/100 g body weight)	1.5 ± 0.1	1.5 ± 0.1	1.7 ± 0.1	1.5 ± 0.1
Heart (g/100 g body weight)	0.5 ± 0.1	0.6 ± 0.1	0.6 ± 0.1	0.6 ± 0.1
Lung (g/100 g body weight)	0.8 ± 0.1	0.8 ± 0.1	0.7 ± 0.1	0.8 ± 0.1

Beginning 1 day after tumor implantation, the animals were treated intraperitoneally for 21 consecutive days. The negative control (CTL) was treated with a vehicle (5% DMSO) used to dilute EO. 5-Fluorouracil (5-FU, 10 mg/kg) was used as a positive control. The data are presented as the mean ± S.E.M. of 10–20 animals.

**Table 5:** Effect of *Cyperus articulatus* rhizome essential oil (EO) on the hematological parameters of peripheral blood samples from C.B-17 SCID mice with HepG2 cell xenografts.

Parameters	CTL	5-FU	EO	
Dose (mg/kg)	-	10	40	80
Erythrocytes (10^6^/mm^3^)	5.2 ± 1.1	7.6 ± 0.8	5.4 ± 1.1	6.7 ± 1.3
Hemoglobin (g/dL)	21.2 ± 4.8	17.7 ± 3.1	26.8 ± 0.7	18.0 ± 1.4
MCV (fL)	43.8 ± 0.4	45.0 ± 3.0	41.5 ± 0.5	44.8 ± 0.5
Platelets (10^3^/mm^3^)	247.2 ± 38.5	222.1 ± 41.6	456.7 ± 112.2	464.1 ± 62.4
Leukocytes (10^3^/mm^3^)	5.2 ± 0.8	2.5 ± 0.6	7.6 ± 0.7	2.9 ± 0.7
Differential leukocytes (%)				
Granulocytes	24.1	28.4	25.7	34.3
Lymphocytes	41.5	46.1	52.2	40.4
Monocytes	33.6	25.5	21.2	25.3

Beginning 1 day after tumor implantation, the animals were treated intraperitoneally for 21 consecutive days. The negative control (CTL) was treated with a vehicle (5% DMSO) used to dilute EO. 5-Fluorouracil (5-FU, 10 mg/kg) was used as a positive control. The data are presented as the mean ± S.E.M. of 7-14 animals. MCV: Mean Corpuscular Volume.
